# Systematic structural discrepancy assessment for computer models

**DOI:** 10.1098/rsta.2024.0214

**Published:** 2025-04-02

**Authors:** Michael Goldstein, Ian Vernon, Jonathan A. Cumming

**Affiliations:** ^1^ Department of Mathematical Sciences, Durham University, Durham, UK

**Keywords:** emulation, computer models, discrepancy, model inadequacy

## Abstract

Model or structural discrepancy is an essential component in the analysis of computer simulators, representing the differences between the outputs of the simulator and the real-world system that the simulator seeks to represent. This discrepancy can arise from various sources such as simplifications of the model science in the simulator, choices made in our particular implementation of that science, and epistemic uncertainties such as the absence of features or science that we did not know to include or have yet to discover. In this paper, we define and distinguish two types of discrepancy: internal discrepancy that can be assessed by experiments on the simulator itself; and external discrepancy which lies outside the scope of such experiments. We present a tractable methodology and workflow for the assessment of structural discrepancy on the basis of collections of experiments applied to the computer model and illustrate our approach in the context of a simple biological model.

This article is part of the theme issue ‘Uncertainty quantification for healthcare and biological systems (Part 2)’ .

## 1. Introduction

Suppose that we have a simulator for a physical system, that we have observations of the real-world system against which to compare the simulator and that we wish to make statements about the real world using this information. No matter how complex a simulation model of a physical process is, there will always be differences between the outputs of the simulator and the real process that the model is intended to represent. Inevitably, there will be simplifications in the model science based on features that are too complicated for us to include, features that we do not know that we should include, mismatches between the scales on which the model and the system operate, simplifications and approximations in solving the equations determining the system, and so on.

If we are interested in making statements about the real system using results from the simulator, we must incorporate these differences into our analysis. Failure to do so leads to grossly inaccurate inferences, such as overfitting of the model to historical data, wrongly ruling out potentially useful models and overconfidence in subsequent predictions [1].

Informally, model or structural discrepancy is the difference between reality and appropriately chosen simulator output. As we will be uncertain about this difference, it is natural to express our knowledge probabilistically, to be incorporated, for example, within a Bayesian analysis. This article is concerned with systematic methods to quantify our knowledge about structural discrepancy in a form that we can use to make inferences about the real physical process. In particular, we will emphasize the value of separation of structural discrepancy into internal and external components, corresponding to features which may be assessed by computer experiments and features which lie outside such experiments, and explain the role of emulation for integrating each aspect of the discrepancy assessment within a manageable workflow.

## 2. Specifying model discrepancy

Suppose that we have a model M(⋅) for a physical system. The model takes as inputs a vector x related to system properties and outputs a vector M(x) representing some features y of the behaviour of the physical system. The model is implemented as a computer simulator f(x) . Often, we have historical observations, z, made, with error, on a subset, ${\underline{y}}_{h}$ , of the elements of y, with corresponding functional outputs fh(x) .

Sometimes, we may consider that there is a unique ‘true’ or ‘best’ choice, x* , for the input vector x , and we use the observed value of z to make an inference for this value, for example assessing a Bayesian posterior distribution for x* . This process is termed *calibration* [2]. At other times, we do not consider that there is such a unique true value, for example if the inputs are tuning parameters. In such cases, we may wish to identify the collection of all input choices *x* for which the simulator is able to reproduce the observed system history. By this, we mean that the outputs fh(x) are acceptably close to the observed values of z , when we have taken into account all of the uncertainties relevant for assessing the quality of the match. This process is termed *history matching* [3,4].

To carry out either of these procedures, we need a probabilistic representation of the difference between the simulator and the physical system, which we can evaluate for each choice of x . A very common way to introduce such model discrepancy is by supposing that, if x* is an appropriate choice for x , then the state of the world, y , is given by


(2.1) y=f⁣(x*)+ϵd,

where $\epsilon_{d}$ is a random ‘error’ vector capturing the deficiencies and missing features of the model, and which is independent of everything else. This choice has the great virtue of simplicity—we just add, say, a 10% error to all uncertainty statements about the real world produced by the simulator.

This is far better than ignoring discrepancy altogether, but we usually can, and should, be more careful. For example, suppose that the main reason for structural discrepancy is that the implementation of the model as a simulator involves certain approximations for the solutions of the underlying system equations. Suppose further that for some input choices these approximations have negligible effects whereas for other choices they have substantial effects. Then acceptable history matches for the former choices would require a closer fit to the data than matches for the latter choices, and similarly the likelihood for a Bayesian calibration would need to include an additional component of uncertainty for those input choices with larger model implementation errors.

The difference between a model and the corresponding physical system is a complex structure which has many different possible representations. We will suggest general forms which are sufficient to support many different aspects of structural discrepancy. Much of our description relies on the construction of emulators for elements of structural discrepancy. Emulators are a familiar feature of uncertainty quantification problems. Often, in practice, we are only able to evaluate f for a limited number of values of x , because of time and resource constraints. In such cases, we usually construct emulators for the elements of f itself.

An *emulator* for a function is a fast surrogate model for the function, with known uncertainty of approximation, which allows us to carry out detailed exploration of model behaviour [2,3]. A common form for such an emulator, for an individual component fi(x) of f , which will be sufficient for our discussion, is to represent the function as


(2.2) $$f_{i}(\underline{x})=\sum_{j}a_{ij}g_{ij}(\underline{x})+u_{i}(\underline{x}),$$

where $a_{ij}$ are constants to determine, gij(x) are deterministic functions, for example polynomials, of *x*, and ui(x) is a stationary stochastic process, often with E[ui(x)]=0,∀x and a correlation function reflecting the smoothness of the function. For example, a common choice is


$$C\text{orr}\left[u_{i}({\underline{x}}_{1}),u_{i}({\underline{x}}_{2})\right]=e{\;}^{-({\underline{x}}_{1}-{\underline{x}}_{2}{)}^{T}}\Sigma ({\underline{x}}_{1}-{\underline{x}}_{2}),$$

where Σ is an appropriate scaling matrix. We may specify a complete probabilistic form for ui(x) , for example a Gaussian process, for a standard Bayesian calibration analysis. Alternately, for history matching, we only require means and variances, so that we can specify only the first and second moments of *u*, as this is sufficient for a Bayes linear analysis [3,5].

In what follows, we will assume that the computer simulator is represented by a corresponding emulator and explain how the notion of structural discrepancy is represented by appropriate modifications to this emulator. In order to do this, we will structure such discrepancy by separating it into two categories, namely *internal* and *external* model discrepancy.

Internal discrepancy arises from the specific technical choices made in the implementation of the model as the current simulator, f , and can be assessed by direct experiments on the simulator itself. Therefore, we learn about internal discrepancy by performing such experiments. External discrepancy comprises all of the features arising from limitations of the modelling process and which, therefore, cannot be quantified through such simulator experiments. Some of these features may correspond to processes that we know we have omitted from the model. Others may come from our recognition of the limitations of our understanding about the processes underlying the model. Finally, some may arise simply as we lack the time, expertise or resource to carry out the appropriate computer experiments, and thus we must count such aspects of structural discrepancy as external as well.

We will now discuss each of these two forms of discrepancy.

## 3. Internal discrepancy

### (a) Internal discrepancy experiments

Internal discrepancy refers to any aspect of discrepancy variation that we choose to quantify by direct experiments on the computer simulator. These assess the effect of the various simplifying assumptions made in the model and the simulator implementation. As with any other aspect of modelling, we must choose the level of detail with which we carry out the internal analysis. Care at this stage will reduce the effort required at the later stages when we must account for all other aspects of structural discrepancy which were not included in the internal assessment. Here are some examples.

1. We may vary parameters that are usually held fixed at pre-assigned values in the planned evaluations of the simulator, where this choice has been made in order to reduce dimension for the input space.
2. We may have several subgroups, for example classified by gender, age and so forth, where the simulator uses the same parameter input values for each subgroup, for reasons of simplicity and ease of model fitting. The simulator may similarly impose coarse partitions for continuous effects, for example young, middle aged and old for effects which are continuous in age.
3. We can add extra flexibility to our input parametrization, for example allowing some constant parameter values to vary over time and space.
4. If the model science is based on the deterministic propagation of a state vector, then we may add a small amount of random noise at each propagation step, in recognition that the model rules oversimplify the true propagation of the state vector.
5. If the model uses a simplified solver, then we may explore the effect of more careful equation solvers, for example increasing the grid resolution and number of iterations for the solver.
6. The simulator might use fixed initial conditions, boundary conditions or external forcing functions, which we might choose to vary.

The aim of these experiments is not to fit a more complex model, as we judge that would be too expensive to fit to data or for real-world use. Instead, we quantify the effect of making the collection of simplifying assumptions given our intention to use our original simulator. However, if the experiments do identify such features as very large discrepancy bias or variance, then we would want to identify which features of the experiments were the major causes of these effects and consider modifying the simulator accordingly.

In each case, our ability to vary the corresponding feature depends in part on how the simulator is coded. Varying fixed parameters, for example, will usually be straightforward. Adding noise to the state vector depends entirely on whether this vector is accessible to access and modify in the simulator code. As a general design principle, the implementation of the model as a simulator should take careful account of the internal discrepancy experiments that we will wish to carry out in order to ensure that the simulator is fit for real-world use.

### (b) Assessing internal discrepancy

We assess internal discrepancy through our chosen experiments as follows.

1. For a single input parameter choice x with model run $f_{,}(\underline{x})=(f_{1}(\underline{x}),$ $f_{2}(\underline{x}),$ …) , we choose a set of perturbations $d_{1},d_{2},...,d_{k}$ , where each $d_{i}$ is a perturbation of each one of the attributes selected for the experiment. We are treating discrepancy judgements as part of the modelling process. Therefore, the perturbations are chosen to reflect the degree of uncertainty that the modeller wishes to introduce. For example, random choices for a parameter value usually held fixed are made by consideration of the level of variation that is thought appropriate to introduce for the parameter. Similarly, we may replace a parameter fixed over time with the output of a continuous stochastic process, with mean equal to the parameter value, small variance and high correlation to represent the modified parameter at each time point, for example by judging the anticipated amount of drift in the parameter over a choice of fixed time intervals that would be considered acceptable and choosing parameters of the process to match these conditional judgements. As with any modelling process, we may explore different choices for such effects, for example choosing sets of samples representing different levels of variation.
2. We evaluate the collection of k model runs, $F_{,}(\underline{x})=(f_{,}(\underline{x},d_{1}),$ $f_{,}(\underline{x},d_{2}),$ …, $f_{,}(\underline{x},d_{k}))$ . $F_{,}(\underline{x})$ is a sample from the internal discrepancy distribution at input $\underline{x}$ .
3. For each output, $f_{r}(\underline{x})$ , we look at the empirical distribution of $F_{r}(\underline{x})$ , from which we may choose to extract simple summary statistics. Natural choices are the bias and variance:

(3.1)Br(x_)=fr(x_)−1k∑i=1kfr(x_,di),(3.2)Vr(x_)=1k−1∑j=1k[fr(x_,dj)−1k∑i=1kfr(x_,di)]2.

- We repeat this experiment for a range of input choices x1,…,xn , giving discrepancy samples F⁣(x1),...,F⁣(xn) . If the sample distributions are very similar, for each xi , then the average is a practical working choice for internal discrepancy.
- Otherwise, we extract summary statistics, for each xi , and build emulators for our chosen discrepancy summaries, for example B⁣(x) and $V_{,}(x)$ , across the input space. Often this emulation is easy as these turn out to be simple smooth monotonic functions. If the number of experiment repetitions, k , is large, then we can view the observed sample statistics as being equal to the underlying population values for each chosen input value. Otherwise, we consider the sample statistics as estimates of these population values and add appropriate standard errors of estimation for our uncertainty about each population value, given the sample. This is similar to the standard way in which we build emulators for summary measures, such as the mean response, for stochastic simulators [6].
- We now have a value, with estimated uncertainty, for the internal discrepancy, at each choice x in the input space. Further, each internal discrepancy experiment gives samples from the full joint distribution of the internal discrepancy variables for all of the outputs. Therefore, we can compute any sample summaries for the joint distribution that we need; for example, we can assess the sample correlation between any output pair Fr(xi) and Fs(xi) for each xi .
- We now choose a form to incorporate internal discrepancy into our emulator for f . If we have been mainly focusing on bias and variance, then a simple representation would be of the form
(3.3) fI(x)=f⁣(x)+μI(x)+σI(x)ϵ,
  where μI(x) is a vector of bias terms and σI(x) is a vector of scale parameters, dependent on x , given by the emulators built from the internal discrepancy experiments. We may view ϵ as a vector with zero mean and unit variance, independent of everything else, with correlation structure based on a combination of the corresponding correlation structures evaluated by the internal discrepancy experiments. If these vary greatly, we may take more care and emulate the general structure of this correlation matrix.

## 4. External discrepancy

External discrepancy arises from the inherent limitations of the modelling process embodied in the simulator and cannot be assessed by simulator experiments. This adds onto the internal discrepancy as


(4.1) f⁣*(x)=MfI(x)+ϵE(x).

Here, $\epsilon_{E}(x)$ has some stochastic specification, given x , as we will describe, and M is a scaling matrix, typically diagonal and often the identity, which allows us to rescale the simulator output to adjust for any mismatch between the scales of the simulator and the real-world phenomena.

One way to analyse the external component of structural discrepancy is described in [7] and is as follows. The function f describes how system properties (the inputs) affect system behaviour (the outputs). Our simulator approximates both the properties of the system and the rules for assessing system behaviour given system properties. Therefore, we may consider that our simulator is an approximation to a more detailed form, $f^{*}$ , sometimes called the *reified model* (from *reify*—to consider an abstract concept to be real). $f^{*}$ embodies all of our judgements about refinements to the science, improvements to solution accuracy, etc., so that additional structural discrepancy on top of this model will be unstructured.

We apply the simple discrepancy model (2.1) to $f^{*}$ and to $f^{*}$ alone, i.e.


(4.2) $$\underline{y}=f_{,}^{*}({\underline{x}}^{*},{\underline{w}}^{*})+\epsilon^{*},$$

where $\epsilon^{*}$ is independent of everything else and w* are any extra model parameters that we might introduce for the reified form.

In this construction, the model, f , is informative for the actual system, y , because f is informative for reified model $f^{*}$ . We cannot evaluate $f^{*}$ but we can emulate it, so all of the analyses (history matching, calibration, forecasting) that we habitually carry out using the emulator of f can be transferred directly to the emulator of the reified form.

To see how we might emulate $f^{*}$ , consider the simplest case, with one input x and one output $f_{,}(x)$ , and an emulator for f of form $f_{,}(x)=a+bx+\epsilon (x)$ , where a,b are constants and ϵ(x) is a stationary stochastic process. A simple emulator for $f^{*}$ might be $f_{,}^{*}(x)=a^{*}+b^{*}x+\epsilon^{*}(x)$ , where $a^{*},b^{*}$ are uncertain constants (with prior means a,b ) and $\epsilon^{*}$ is a stationary process correlated with ϵ ; for example we might set $\epsilon^{*}(x)=\gamma \epsilon (x)+\delta \epsilon^{\prime }(x)$ , where ϵ and $\epsilon^{\prime }$ are independent. This expresses our judgement that, with more careful modelling, the global form of the emulator will not change but the rate of change of $f_{,}(x)$ with x is very likely to change. This form allows us to express structured judgements as to the potential effects of more detailed modelling. In contrast, the simple form for discrepancy, (2.1), is equivalent to imposing the simplified version of the reified emulator of form $f_{,}^{*}(x)=a^{*}+bx+\epsilon (x)$ .

This approach is termed *direct* reification. In more generality, suppose that our emulator for component i of f is of form (2.2). Then our simplest emulator for $f^{*}$ would then be


(4.3) fi*(x,w)=∑jaij*gij(x)+ui*(x)+ui**(x,w),

where we might model $a_{ij}^{*}$ as $a_{ij}^{*}=c_{ij}a_{ij}+\nu_{ij}$ with, for example, $c_{ij}$ treated as known, reflecting modelling judgements as to the relative rates of change of the two functions (often we will set these to one), and $\nu_{ij}$ treated as uncertain. We may choose to correlate u(x) and u*(x) , if we consider that divergences from the global form will share common features, but we will usually leave u**(x,w) uncorrelated.

If we have more detailed judgements about particular deficiencies in the simulator, then we may build an emulator $f^{\prime }$ which represents the particular effects that we are considering and then link $f^{\prime }$ to $f^{*}$ through direct reification. This is termed *structural* reification. For a discussion of the assessment of all of these quantities and an example of structural reification, see [7], which illustrates each part of this assessment in the context of a compartmental model for the potential shutdown of the Thermohaline circulation in the Atlantic ocean, by adding a notional additional compartment to represent aspects of circulation not captured in the given model and assessing the effect of this modified flow on each component of the model.

## 5. Structural discrepancy workflow

Given data, z , corresponding to simulator outputs fh(x) , we are usually interested in structural discrepancy mainly for those input choices which give acceptable matches to z . In such cases, we can greatly simplify the workflow for discrepancy quantification. First, we identify, by history matching, the subspace of input values for which the simulator output is sufficiently close to the observed system history to be of interest as a possible choice for real-world uses of the simulator. We can use a simple and cautious overall order of magnitude discrepancy assessment for this purpose. A good software package for carrying out such history matching is HMER [8]. Then, we re-sample and re-emulate the simulator, carry out the internal discrepancy experiments and derive discrepancy forms, such as (3.3) and (4.3), all within this input subspace. This will often be a much simpler task than to emulate and assess discrepancy accurately for the whole of the input space, partly as the new space is much smaller and partly because function behaviour within the reduced space is usually much more consistent. We usually go through a final stage of history matching with the more careful emulators and discrepancy assessments.

Depending on the problem at hand, the history match may be the endpoint of the analysis. Alternately, if we prefer to calibrate the model, for example, if we wish to make inferences about the likely values of some of the model parameters which have clear physical meanings, then we may carry out a full Bayesian analysis within the reduced parameter space using the disrepancy structure that we have constructed within this space.

We may have further goals for our modelling. For example, we may want to forecast, which requires careful structural discrepancy assessment across past and future outcomes for plausible choices of inputs, x* . If we want to predict some future system outcomes, $\underline{y}{,}_{p}$ , corresponding to function outputs fp(x) , given observed historical data z , then we update uncertainties for $\underline{y}{,}_{p}$ given z , for each acceptable choice of x* , using the decompositions


(5.1) $$\begin{array}{rlrl} \underline{z} & =f_{h}({\underline{x}}^{*})+\epsilon_{h}^{*}({\underline{x}}^{*})+e_{h}, & \;\underline{y}{,}_{p} & =f_{p}({\underline{x}}^{*})+\epsilon_{p}^{*}({\underline{x}}^{*}), \end{array}$$

where $\epsilon_{p}^{*},\epsilon_{h}^{*}$ are structural discrepancies for $\underline{y}{,}_{p},\underline{y}{,}_{h}$ and $e_{h}$ is measurement error for z [3]. Where our beliefs are described by a second-order specification, the appropriate mechanism for updating those beliefs are the Bayes linear [5]:


(5.2) $$\begin{array}{rl} E_{\underline{z}}[\underline{y}{,}_{p}] & =E[\underline{y}{,}_{p}]+Cov[\underline{y}{,}_{p},\underline{z}]Var{\left[\underline{z}\right]}^{-1}(\underline{z}-E[\underline{z}]),\\ V{\text{ar}}_{\underline{z}}[\underline{y}{,}_{p}] & =V\text{ar}[\underline{y}{,}_{p}]-Cov[\underline{y}{,}_{p},\underline{z}]Var{\left[\underline{z}\right]}^{-1}Cov[\underline{z},\underline{y}{,}_{p}], \end{array}$$

where $E_{\underline{z}}[\underline{y}{,}_{p}]$ and $V{ar}_{\underline{z}}[\underline{y}{,}_{p}]$ represent the adjusted expectation and variance of the future outcomes, $\underline{y}{,}_{p}$ , given the observations, z - in other words, our forecasts. Where beliefs about z and $\underline{y}{,}_{p}$ are described probabilistically then the distribution $\underline{y}{,}_{p}\;|\;\underline{z}$ is required, commonly obtained with Markov chain Monte Carlo methods.

## 6. Discussion

In this paper, we treat the assessment of structural discrepancy as part of the modelling process. This may be contrasted to approaches based on variants of (2.1); see for example [2] in which model discrepancy depends only on controllable parameters such as time. In such approaches, assessment of model discrepancy becomes a problem of statistical estimation, which raises important technical challenges as there is confounding between the model response and the structural discrepancy component. Various methods have been developed to address these challenges, for example, the modularization approach, introduced in the context of computer models in [9], in which these problems are handled by separating the probabilistic specification into discrete sub-modules and carefully controlling message passing between them. This is similar to the idea of ‘cutting feedback’ as implemented in the popular Bayesian software package Winbugs [10]. A common estimation technique in this area to address such issues, introduced in [11], is based on projection using the $L_{2}$ norm of the function, which can be viewed as a continuous analogue of ordinary least squares methods for parameter choice which minimize squared differences between physical outputs and simulation outputs. A Bayesian formulation for such approaches was introduced in [12]. A recent overview of advancements in addressing the challenges of the unidentifiability issues raised when incorporating model inadequacy, and related issues, is given in [13].

In our formulation, such confounding is not an issue as each part of our uncertainty specification is separately modelled and assessed. Further, the basic issue with any approach based on variants of (2.1) is that it implies there is a value $x^{*}$ for which all of the information that the simulator provides is contained in the single evaluation $f(x^{*})$ . Usually, in practice, this is unlikely to be the case. [7] provides a careful analysis of the potential logical inconsistencies in such a view, for example by considering the thought experiment of constructing an improved simulator which respects the physical behaviour of the system more closely than does the current simulator and demonstrating contradictions within the single sufficient evaluation view. See also [14] for a somewhat more structured approach to model discrepancy assessment, albeit within a fairly specific setting.

## 7. Example: the predator–prey model

**Figure 1.**
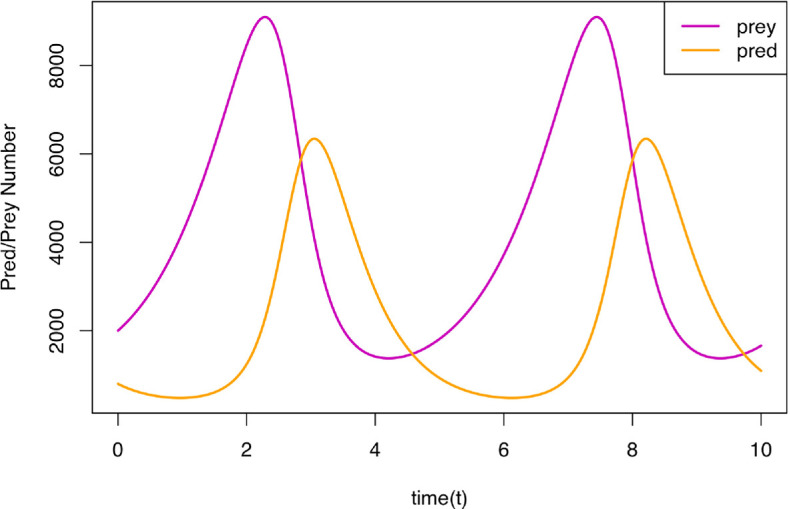
Output from a single run of the Lotka–Volterra model against time. Note that prey (purple line) exhibits the classic peaks in population level, while the predator population (orange lines) has similar peaks that follow that of the prey, with the whole system exhibiting oscillatory behaviour.

To illustrate the methods for assessing model discrepancy, we employ a relatively simple and fast Lotka–Volterra Predator–Prey (LVPP) model. This will allow us to focus on demonstrating the structural discrepancy assessment, however when analysing more expensive and/or more complex models we would follow the same workflow, but make more use of emulation and history matching (to perform a more extensive input parameter search), as discussed below. See [15] for an analysis of a more complex variant of the LVPP model. The LVPP model represents the dynamics, over time, of two interacting species—the prey, $f_{1}$ , and the predators, $f_{2}$ —describing the changing populations of each species over time and generating a two-dimensional time series of species counts, indexed by time t . The system’s dynamics are described by the following pair of differential equations:


(7.1) $$\begin{array}{rlrl} \frac{\text{d}f_{1}}{\text{d}t} & =x_{1}f_{1}-x_{2}f_{1}f_{2}, & \;\frac{\text{d}f_{2}}{\text{d}t} & =x_{2}f_{1}f_{2}-x_{3}f_{2}, \end{array}$$

where x=(x1,x2,x3) are the inputs to the model and comprise three rate parameters that govern the speed of reproduction of prey, the predator–prey interaction and the death rate of predators, respectively. The output from a single evaluation of the Lotka–Volterra model is shown in figure 1, exhibiting the classic lag between peaks of prey and predator populations over time.

**Figure 2.**
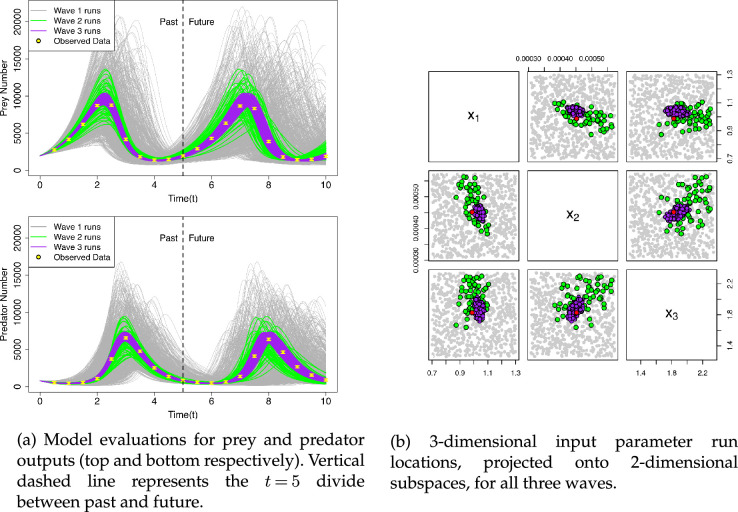
Lotka–Volterra evaluations in output space (left) and input space (right) for wave 1 (grey), wave 2 (green) and wave 3 (purple). Red point (right) is the wave 2 test point. Yellow points and error bars (left) are the observed data.

For our analysis, the input parameters to the model are assumed to have ranges of $x_{1}\in \left[0.7,1.3\right]$ , $x_{2}\in \left[3.1\times 10^{-5},5.7\times 10^{-4}\right]$ and $x_{3}\in \left[1.3,2.3\right]$ , representing partially informed prior information. Note that, wider ranges would require a more detailed multi-wave history match [16], which although perfectly feasible, is not our focus here. The LVPP equation (7.1) can be numerically integrated to generate a time series of simulator output once the initial conditions have been specified, which we here take to be $[f_{1}(t=0),f_{2}(t=0)]=\left[2000,800\right]$ . To explore the full ranges of input parameter values, a first *wave* of 750 runs of the model were designed over the three-dimensional input space using a maximin Latin hypercube [17], and the collection of resulting model outputs are drawn as the grey curves in figure 2a, with their corresponding input values shown as grey points in figure 2b.

To illustrate our structural discrepancy assessment workflow for this model we require observed data, z . As this model is entirely synthetic, pseudo-observations are generated from a single evaluation of a more complex and stochastic version of the Lotka–Volterra model simulated using the Gillespie algorithm (see e.g. [18]), under slightly different initial conditions (1910,710) , and using time-varying inputs x→g(t)x that vary with a quadratic dependence centred around 1: $g(t)=1-((t-5{)}^{2}$ /25−0.5)/10 , representing a subtle seasonal, periodic change to the reaction rates of the real system. We, therefore, obtain a set of system outputs y that cannot be perfectly recreated using the LVPP model described above—just as would be expected in any analysis of a real-world system and its corresponding data. Uncorrelated observation error e with σe=50 (representing the accuracy of the observation process) is added to obtain pseudo-observational data z=y+e , and is shown in figure 2a as yellow points and error bars.

### (a) Initial history matching

Given the data z , we are interested in assessing the structural discrepancy of our simulator for input choices that give acceptable matches to the observations. However, a cursory inspection of the model evaluations in figure 2a reveals that a sizeable majority of the first wave of model evaluations show little to no correspondence to our observations. Thus, we follow the outlined workflow and begin by seeking to refine our initially broad and conservative input space to a more focused set of parameter combinations which could feasibly yield outputs that are a close enough match to the data to be of further interest. The methodology here is that of history matching [6,19] using a conservative order of magnitude model discrepancy assessment of ±15% of model output values. Our model discrepancy choice is deliberately cautious at this stage and will be refined in the subsequent analysis; its purpose here is to simply ensure that obviously incompatible input parameter combinations can be readily identified and removed from consideration. We anticipate that the refined assessment will be smaller than this initial, cautious ±15% value. Given this discrepancy and simple univariate emulators for the simulator outputs, we compute the *implausibility*, I(x) , at each of our wave 1 input points:


(7.2) I(x)=|E[f⁣(x)]−z|Var[f⁣(x)−z]=|E[f⁣(x)]+E[ϵh*]−z|Var[f⁣(x)]+Var[ϵh*]+Var[eh],

where E[f⁣(x)] and Var[f⁣(x)] are the expectation and variance of our emulator of the simulation at x (required for slow models while here we use the model output directly), z is the observed data, $e_{h}$ is the observational data error and $\epsilon_{h}^{*}$ is our structural model discrepancy. This yields an implausibility value for every input parameter choice, x , and every output component of y . The implausibility measure takes large values in the presence of strong disagreement between a particular simulator output and its corresponding data, and small values in the presence of either good matches or high uncertainty [19]. These implausibility measures are then combined into a single implausibility value, for each input point x , via the maximum implausibility:


(7.3) IM(x)=maxi⁡I(i)(x),

where I(i)(x) is the implausibility (7.2) calculated for the i th output component of the simulator. This ensures that if the simulator fails to match the data on any single component, it is judged an implausible match. Conversely, good matches are only declared when all output components have correspondingly small implausibilities and so are close to the data with low uncertainty for all model outputs. Here, to demonstrate our methods, we take the historical data z to be outputs from the first peak only, such that 0≤t≤5 . The time t=5 is viewed as the ‘present day’ (shown as the vertical dashed line in figure 2a) and all other future data for t>5 is shown for comparative purposes but not used in the history match, nor in the subsequent forecasts.

To distinguish these implausible input points to exclude from future study from the remaining non-implausible set of points, we apply a threshold to the maximum implausibility computed via (7.3) and retain all those input points with lower implausibilities as our *wave 2* input points. We choose a threshold of IM(x)=3 motivated by Pukelsheim’s 3-sigma rule [20] which states that for any uni-modal continuous distribution, 95%of its probability lies within 3-sigma of its mean. When applied directly to the 750 runs, this results in 60 suitable model runs that are deemed sufficiently compatible with the observed data to warrant further analysis. These are shown as green lines in figure 2a and in the context of the input space in figure 2b as green points, which now more closely mirror the observed data over the first peak ( 0≤t≤5 ) and correspond to a smaller region within the input parameter space. This refinement stage can be repeated multiple times with additional design points generated in this reduced input space and emulators re-fit after each wave to focus further if required. See [16,21,22] for details regarding history matching, including applications in cosmology, systems biology and epidemiology and also for comparisons to alternative approaches such as MCMC and ABC.

### (b) Internal discrepancy assessment

We perform the internal model discrepancy assessment following the steps of §3(b). We begin with a single input location chosen from our wave 2 runs (step (i)), hereinafter referred to as the *wave 2 test point* and highlighted in red on figure 2b. We select a set of perturbations $d_{1},\ldots ,d_{k}$ of the model inputs to be applied to the test point, in this case formed by varying initial conditions and—given the time series nature of this simulation—adding temporal variation to the inputs. Specifically, for each perturbation, the initial conditions were drawn from $N({2000,40}^{2})$ and $N(800,40^{2})$ for prey and predator, respectively, rather than being fixed at the values of 2000 and 800 that were used in the main model runs, with the SD of 40 chosen to reflect the scientist’s uncertainty over these previously fixed initial conditions. Time-varying inputs were introduced by modifying the LVPP equation (7.1) to multiply the rate constants by functions of time, such that each $x_{i}$ is replaced by $g_{i}(t)x_{i}$ for i=1,2,3 . The function $g_{i}(x)$ was chosen to be cyclical about the value of 1 and took the form


(7.4) $$g_{i}(t)=1+c_{i}\sin\left[(2\pi \{t-a_{i}\}b_{i})/T\right],$$

where T=10 , and where $a_{i}∼U(4.5,5.5)$ , $b_{i}∼U(0.6,0.9)$ and $c_{i}∼N({0.02,0.05}^{2})$ are drawn independently for each perturbation. Note that the time variation here is sinusoidal and hence different from the quadratic variation used to generate the real system y . Hence, it along with the distributional choices for $a_{i},b_{i}$ and $c_{i}$ is designed to capture the scientist’s uncertain judgements regarding a possible subtle seasonal effect (any remaining uncertainty, not captured by this parameterised form, can be included in theexternal discrepancy). Following this scheme, we construct 50 perturbations for each of the initial conditions and the time-varying inputs separately, as well as 200 perturbations where both elements were varied together in order to assess the impact of each modification and compare their relative effects.

**Figure 3.**
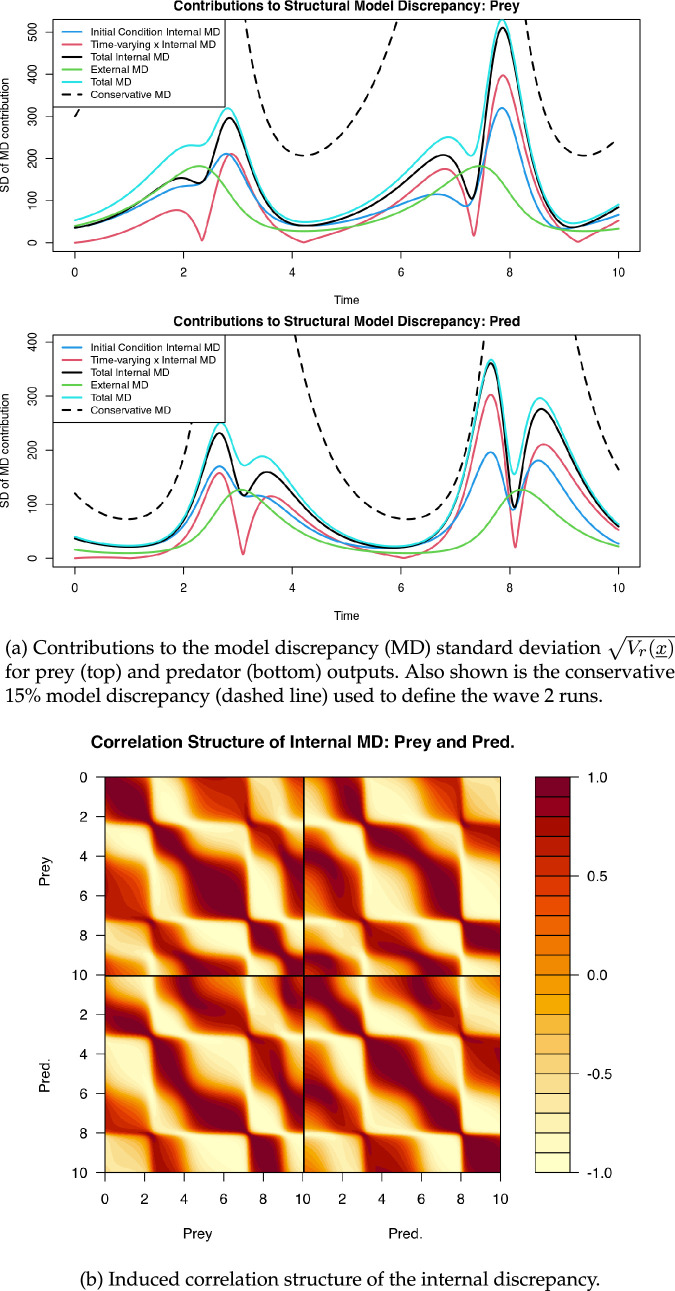
Results of internal discrepancy analysis at the wave 2 test input point.

We now evaluate the collection of simulator runs under each of our permutations, F⁣(x)=[f⁣(x,d1), f⁣(x,d2), …, f⁣(x,dk)] , per step (ii). The resulting collection of evaluations form a sample of the internal discrepancy distribution, which can be summarised (step iii) by simple statistics such as bias (3.1) and variance (3.2). In figure 3a, we focus on the latter, where we plot the contributions of the initial conditions and the time-varying inputs to the standard deviation of the internal discrepancy, i.e. Vr(x) . The contributions to the internal discrepancy due to varying initial conditions and time varying x inputs are shown as the blue and red lines, respectively (calculated using the two sets of 50 perturbations that varied each internal feature), while the total internal discrepancy Vr(x) is given by the black line (calculated using the 200 perturbations that varied both internal features). A nominal external discrepancy equal to 2% of the model output is employed to represent the remaining structural model discrepancy not captured by the internal analysis and is given by the green line. The total model discrepancy is shown as the light blue line. The conservative model discrepancy used above to define the wave 2 runs ( 15% of the model output) is shown as the dashed black lines.

First, we note that the discrepancy contributions are not uniform, and clearly vary with time with peaks in the vicinity of those observed in the data. The initial condition uncertainty (blue line) is a dominant component of discrepancy at early times, but while the effects of the time-varying inputs (red line) are small at first they occasionally become the dominant source of discrepancy at late times. These results are intuitive, with simulation output most sensitive to initial conditions at early times and becoming less relevant as the simulation progresses. Note also that the total model discrepancy is substantially less than the conservative 15% used in the initial history matching stage, so there is no risk of mistakenly excluding viable parameter combinations.

A particularly insightful feature of this approach to the assessment of internal discrepancy assessment is that we can also examine the correlation structure (across all outputs over time, and of prey and predator type) of the induced internal discrepancy. From the collection of 200 permutations where we varied both sources of discrepancy, we can construct the sample correlation matrix which is shown in figure 3b. This approximates the correlation Corr[Fr(xi),Fs(xi)] , where r and s label both the time and output type (prey or predator). Here, we see substantial structure between the discrepancy over the different output components, with strong positive and negative correlations induced by the coupled and oscillatory behaviour of the simulator outputs. This provides valuable information that would be entirely overlooked were we to take a naive and unstructured model discrepancy specification.

While we can glean substantial information from this analysis alone, we have thus far only explored a single point from the wave 2 runs, and it is reasonable to suspect that the observed internal discrepancy properties may vary with x . Therefore, the natural progression of the analysis is to repeat the permutation experiment with each of the remaining wave 2 input points (step iv).

**Figure 4.**
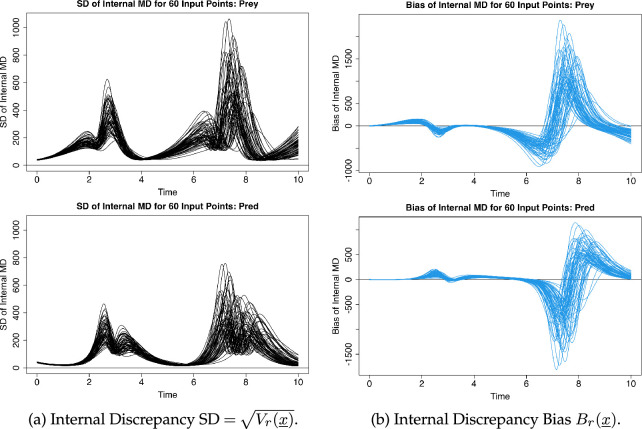
The standard deviation $\sqrt{V_{r}(\underline{x})}$ and bias $B_{r}(\underline{x})$ of the internal discrepancy, calculated at each of the 60 wave 2 input points (giving each of the 60 black/blue lines), plotted against time ( r=t ) for the prey and predator outputs.

Recalculating the internal model discrepancy standard deviation, Vr(x) , for each of the 60 cases we obtain the results in figure 4a. Similar calculation of the biases, Br(x) , yield the results in figure 4b. Each curve in the plots represent the results of the same calculation as that presented in figure 3a, only now applied to each of the wave 2 input points. We can see clearly that both Vr(x) and Br(x) vary substantially with input location x , with similar overall shapes to those observed above albeit with notable variation in magnitude of the peaks. Given that these expressions of the discrepancy clearly vary as the inputs to the simulator vary, it would be inappropriate to simply reduce these results to a simple summary such as an average. If we were to do so, we would grossly oversimplify our assessment of our internal model discrepancy. Instead, we proceed to construct emulators for these quantities over the wave 2 input space (step v) for use in subsequent calculations.

The discrepancy standard deviation, Vr(x) , and bias, Br(x) , were then emulated over the wave 2 locations. Simple emulators based on linear regressions were used, noting that the regression $R^{2}$ was above 0.85 for all outputs. This provides a more detailed and nuanced description of model discrepancy over the space of x that we use to revisit and refine our collection of feasible input points. We can now refine our emulator for the model (step vii) into the form (3.3) by using the emulators of the internal discrepancy bias and standard deviation as the μI(x) and σI(x) components. This provides a more detailed and structured description of the internal discrepancy over the input space, which can be reintroduced to our history matching calculations in §7*a*. By doing so, we can further refine our collection of suitable input parameter combinations using the updated implausibility given the additional information gained during the structural discrepancy analysis. At this stage, we could generate new input candidate points and, by rejection sampling, retain only those which satisfied our implausibility threshold under the new model discrepancy. Doing so yields the new input combinations shown in purple on figure 2b, which could form a potential wave 3 of our analysis were we to continue iterating the history match.

### (c) Using structural discrepancy to enhance forecasting

In the previous section, we carefully assessed the internal model discrepancy via a series of experiments on the computer simulator. While this has already refined our understanding of the gap between our simulation and the real system, the same information can greatly aid other calculations we may seek to perform—such as forecasting, as discussed in §5. Suppose our rationale for exploring this simulation is to predict the next peak population (in terms of time and magnitude) for both species on the basis of only the data observed until time t=5 , with data beyond this point as yet unseen. Specifically, let us consider how we can use information on the location of the first population peak alongside our improved discrepancy specification to update the model discrepancy and improve potential forecasts for the second peak in the two populations.

First, our focus shifts from the time series outputs of the simulator, f⁣(x,t)=[f1(x,t),f2(x,t)] , for the two species, to derived quantities of the timing and magnitude of the k th peak in the population for the i th species, denoted by f~(k)(x)=[f~1,time(k)(x),f~1,mag(k)(x),f~2,time(k)(x),f~2,mag(k)(x)] . Thus from every evaluation of the simulator, we can determine the properties of the first simulated population peak, f~(1)(x) , and the second peak, f~(2)(x) . In the notation of (5.1), our historical simulator outcomes are fh(x)=f~(1)(x) , and our future outcomes to be predicted are fp(x)=f~(2)(x) , and any forecasting calculation will require a structural discrepancy component for both the past, $\epsilon_{h}^{*}$ , and future values, $\epsilon_{p}^{*}$ . Before assessing the internal discrepancy, we begin by specifying a simple zero mean uncorrelated external discrepancy of 2% and ±0.03 for the magnitude and the timing of the peaks, respectively, for both species. Additionally, we adopt a zero-mean uncorrelated observational error $e_{h}$ with standard deviations 50 and 0.025 for the magnitude and timing of the peaks, representing an imperfect peak observation process.

A simple approach to the prediction problem would be to identify a single input choice, x , that we viewed as an acceptable candidate for x* and to explore the forecast this particular choice would give. A naive first step would simply use the simulator output for the second peak, f~(2)(x) , as the forecast, supplemented by the additional uncertainties we had specified for our discrepancies, $\epsilon_{p}$ . However, there are various correlations present between our model discrepancy components - between peaks of predator and prey, and between past and future—which mean we can transfer information about our ability to predict at the first peak to our prediction for the second peak via the model discrepancy. More formally, assuming that x is a suitable x* , the correlations that exist between $\epsilon_{h}$ and $\epsilon_{p}$ induced by the internal discrepancies, in turn, induce correlations between the past observations z (peak 1) and the future system value $\underline{y}{,}_{p}$ (peak 2) via (5.1). These correlations can then be used to update our beliefs about the behaviour of $\underline{y}{,}_{p}$ given z , represented by $E_{\underline{z}}[\underline{y}{,}_{p}]$ and $V{\text{ar}}_{\underline{z}}[\underline{y}{,}_{p}]$ as given by (5.2).

**Figure 5.**
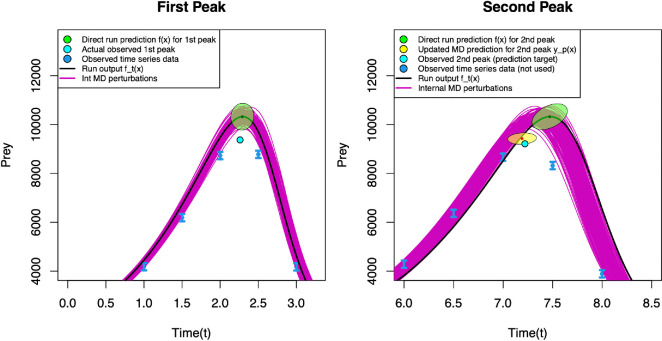
Impact of updating model discrepancy on the second peak location forecast from a single wave 2 evaluation. Left: observed first peak location (light blue point) used to update the model discrepancy. Right: forecast before update (green ellipse) and after update (yellow ellipse). Actual second peak location (the target for the forecast): light blue point. Note that, time series data for the second peak was not used, and is just shown for comparative purposes.

In figure 5, we show an example of this calculation for a single wave 2 input combination, where the model output, f⁣(x,t) , is given as the solid black line and its 200 perturbations from §7*b* indicated as purple lines. For each member of this collection of 200 evaluations F⁣(x) , we can extract the 8 peak outputs of interest, [f~(1)(x),f~(2)(x)] , and generate a 200×8 matrix of simulator outputs from which we can assess the 8×8 discrepancy covariance matrix, V⁣(x) , using (3.2) from §7*b*. This provides valuable information on the discrepancy correlations between all of the 8 peak outputs—the first four of which correspond to the first peak, f~(1)(x) , and the remainder to the second peak, f~(2)(x) . Combining this internal discrepancy information with the unstructured external discrepancy specification gives an assessment of the overall discrepancy variance for the 8 peak output variables.

Using this information, we draw the green ellipses in figure 5 to represent a naive prediction that is centred on f⁣(x) with the ellipse orientation and axis lengths describing the assessed discrepancy uncertainties represented by $V\text{ar}[\epsilon_{h}]$ and $V\text{ar}[\epsilon_{p}]$ . It is clear from the left panel that this yields an inadequate prediction to the first peak, as the observation (cyan point) is both lower and slightly earlier than this simple simulator-based forecast suggests. Therefore, it is reasonable to expect a similar deficiency in our forecast of the second peak using this particular evaluation of the simulator. Using what we have learned about the prediction at the first peak, we can adjust our discrepancy, $\epsilon_{p}$ , and hence our prediction for $\underline{y}{,}_{p}$ to $E_{\underline{z}}[\underline{y}{,}_{p}]$ via (5.2). In the right panel of figure 5, the original naive prediction in green has been adjusted to give the improved forecast for $\underline{y}{,}_{p}$ , $E_{\underline{z}}[\underline{y}{,}_{p}]$ , as the red point, with associated uncertainty, $V{\text{ar}}_{\underline{z}}[\underline{y}{,}_{p}]$ , in yellow, giving a more accurate prediction of the second peak location after this update. It is important to note that this calculation is looking only at a single input choice, x , effectively assuming it is a good candidate for x* . This will not be universally true for all x , and this calculation will not rescue a bad prediction made from an inappropriate input choice; instead the input should be deemed implausible and discarded after a later history match that used the second peak observation.

**Figure 6.**
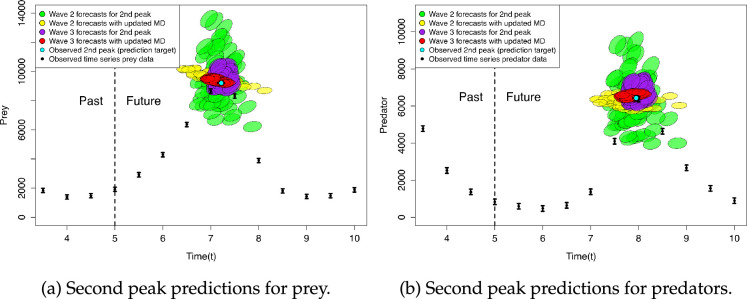
Forecasts for the second peak of the LVPP model: wave 2 predictions without updating the model discrepancy (green), and after updating model discrepancy (yellow); wave 3 predictions without updating the model discrepancy (purple), and after updating model discrepancy (red). Actual second peak location (the target for the forecast): light blue point.

It is instructive to repeat this predictive calculation for all of the cautious wave 2 input points. We thus obtain the results in figure 6 with the simple forecasts for each input as a green ellipse, alongside the corresponding updated forecasts as yellow ellipses. We can clearly see the effect of the updated model discrepancy by substantially reducing the uncertainty around each of the forecasts and moving the forecasts closer to the prediction target. The set of yellow ellipses already deliver a vastly improved forecast for the peaks and represents a somewhat robust forecast as it is based on a very cautious set of wave 2 runs (that were defined using a conservative, large initial model discrepancy assessment). The substantial impact of an additional wave of history matching to refine our space of plausible input parameters can be seen by contrasting these results with the naive (purple) and adjusted (red) predictions using the wave 3 evaluations, which have greatly reduced uncertainties and concentrate predictions in a much tighter region around the prediction target. Refer to figure 2 for the locations and outputs of the wave 2 and 3 runs. We assessed the internal model discrepancy for the wave 3 runs in the same way as for wave 2, but again for a slower model, emulation of the relevant covariance matrices could be employed and would dramatically reduce the total number of runs required.

The above forecasts are in alignment with the history matching paradigm where we do not seek to probabilize the input space and seek to employ only a limited set of uncertainty judgements [16]. However, a further step would be to consider a weighting of the input points used in the forecast according to their fit to data, and thereby weighting the corresponding forecasts they produce. For example, adopting Uniform or Gaussian prior distributions over the inputs lead to tractable forecasts without the need for extensive numerical integration [23]. Alternatively, choices of more general prior distributions effectively leads to a fully Bayesian calibration and forecast [2], though this does require making a number of distributional judgements that may be harder to justify and possibly unnecessary, e.g. if the current forecast dictates a very clear decision choice which will be unaltered by further probabilistic nuance.

## 8. Conclusion

Model or structural discrepancy is an essential component in the analysis of computer models as it reflects the uncertainty that surrounds our simulator’s ability to reproduce the real system it attempts to model. While discrepancy can sometimes be a challenging concept to reason about, we have described various potential strategies for assessing sources of structural discrepancy that are applicable to a wide range of models.

Careful discrepancy assessment will: (i) correct our overconfidence in our projections (by adding appropriate levels of additional uncertainty), (ii) increase our forecast accuracy (by making better choices for x* , avoiding overfitting and correcting for systematic biases in our simulator), (iii) help us to make reliable control choices for future outcomes (by recognising the real-world risks of our various control choices), and (iv) allow us to have a reasoned view as to how the quality of the model affects the quality of forecasts (by modifying features such as the magnitude of discrepancy variances and repeating our calculations).

## Data Availability

Data are simulated, and code to generate data and perform the analysis is available at: https://www.maths.dur.ac.uk/users/i.r.vernon/papers/health_bio/
